# Analysis of Invasive Nontypeable *Haemophilus influenzae* Isolates Reveals Selection for the Expression State of Particular Phase-Variable Lipooligosaccharide Biosynthetic Genes

**DOI:** 10.1128/IAI.00093-19

**Published:** 2019-04-23

**Authors:** Zachary N. Phillips, Charles Brizuela, Amy V. Jennison, Megan Staples, Keith Grimwood, Kate L. Seib, Michael P. Jennings, John M. Atack

**Affiliations:** a Institute for Glycomics, Griffith University, Gold Coast, Queensland, Australia; b Public Health Microbiology, Forensic and Scientific Services, Queensland Department of Health, Brisbane, Queensland, Australia; c Menzies Health Institute Queensland, Griffith University, Gold Coast, Queensland, Australia; d Department of Infectious Diseases, Gold Coast Health, Gold Coast, Queensland, Australia; University of California San Diego School of Medicine

**Keywords:** NTHi, glycosyltransferase, invasive disease, lipooligosaccharide, phase variation

## Abstract

Nontypeable Haemophilus influenzae (NTHi) is a major human pathogen, responsible for several acute and chronic infections of the respiratory tract. The incidence of invasive infections caused by NTHi is increasing worldwide. NTHi is able to colonize the nasopharynx asymptomatically, and the exact change(s) responsible for transition from benign carriage to overt disease is not understood. We have previously reported that phase variation (the rapid and reversible ON-OFF switching of gene expression) of particular lipooligosaccharide (LOS) glycosyltransferases occurs during transition from colonizing the nasopharynx to invading the middle ear. Variation in the structure of the LOS is dependent on the ON/OFF expression status of each of the glycosyltransferases responsible for LOS biosynthesis. In this study, we surveyed a collection of invasive NTHi isolates for ON/OFF expression status of seven phase-variable LOS glycosyltransferases. We report that the expression state of the LOS biosynthetic genes *oafA* ON and *lic2A* OFF shows a correlation with invasive NTHi isolates. We hypothesize that these gene expression changes contribute to the invasive potential of NTHi. OafA expression, which is responsible for the addition of an *O*-acetyl group onto the LOS, has been shown to impart a phenotype of increased serum resistance and may serve as a marker for invasive NTHi.

## Abstract (precis)

Nontypeable Haemophilus influenzae (NTHi) is a major human pathogen, responsible for several acute and chronic infections of the respiratory tract. The incidence of invasive infections caused by NTHi is increasing worldwide.

## INTRODUCTION

Nontypeable Haemophilus influenzae (NTHi) is a clinically significant bacterial pathogen of global relevance. NTHi is able to colonize the human nasopharynx asymptomatically but is also responsible for acute and chronic infections of the respiratory tract, including middle ear infection (otitis media) in children (1), acute exacerbations in protracted bacterial bronchitis, chronic obstructive pulmonary disease and bronchiectasis (2, 3), and community-acquired pneumonia in adults (4). Since the introduction of a vaccine against H. influenzae serotype b (Hib), the incidence of invasive infection caused by NTHi has increased significantly worldwide (5, 6). NTHi is now a major cause of severe invasive disease in neonates and is responsible for invasive infections in children that have significant comorbidities (7, 8). NTHi invasive infections are fatal in ∼10% of children between 2 and 4 years old and in ∼17% of children under the age of 1 (9, 10). The increase in invasive disease caused by NTHi is likely due to multiple factors, including increasing numbers of vulnerable patient populations with complex comorbidities rather than simply Hib vaccine-induced strain replacement (5). Financial and pathological burdens of NTHi are increasing annually in the absence of an NTHi vaccine and amplified by emerging antibiotic-resistant strains (11, 12). Several studies have investigated potential associations between the expression of certain virulence factors and invasive NTHi isolates (8, 13, 14), but none proved conclusive in demonstrating a link between any particular factor and the invasiveness of NTHi.

**FIG 1:**
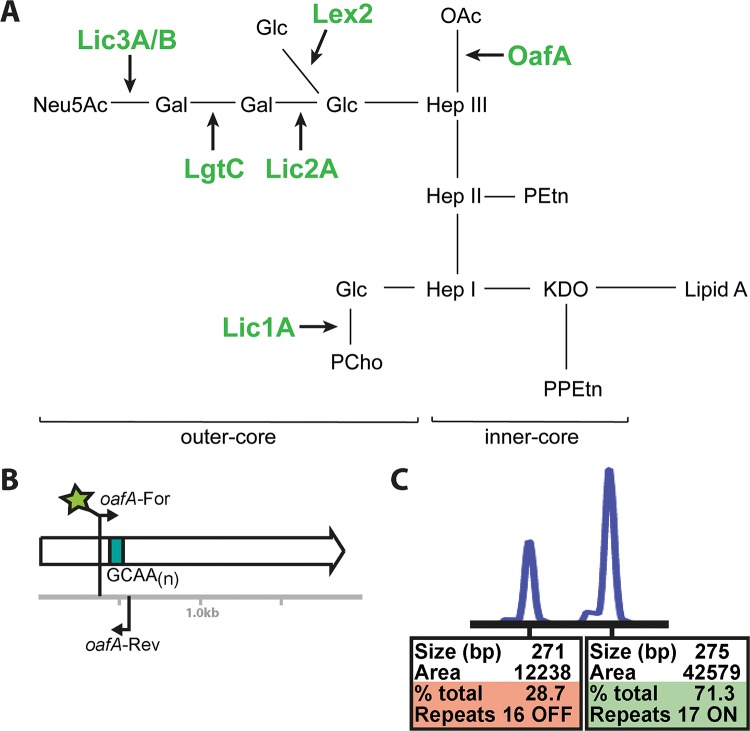
Illustration of NTHi LOS structure and fragment analysis methodology. (A) Schematic representation of NTHi LOS and the roles of the glycosyltransferases encoded by the seven phase-variable loci studied in this work: Lic1A, phosphorylcholine transferase; Lic2A, galactosyltransferase; Lic3A and Lic3B, sialyltransferases; Lex2A, glucosyltransferase; LgtC, galactosyltransferase; OafA, *O*-acetyltransferase (28). NTHi LOS contains 2-keto-3-deoxyoctulosonic acid (KDO), pyrophosphoethanolamine (PPEtn), phosphoethanolamine (PEtn), heptose (Hep), galactose (Gal), glucose (Glc), phosphocholine (PCho), Neu5Ac (*N*-acetylneuraminic acid), and *O*-acetyl group (OAc). LOS structure is therefore dependent on the ON/OFF status of each of these seven genes. (B) An illustration of the PCR technique used to survey the repeat tract length of a phase-variable gene, in this case *oafA*, which contains a variable-length SSR tract made up of a GCAA*_n_* repeat (green box). Primers are designed to bind either side of this repeat tract, with the length of PCR product dependent on the number of GCAA*_n_* repeats present. Therefore, a population will contain a mixture of different-sized PCR products as the length of the repeat tract varies between individual bacterial cells. Fragments are then separated and sized, and the amount of each size was quantified using an ABI GeneScan system by using a fluorescently labeled forward primer (green star). (C) An example of a GeneScan fragment analysis trace, with the area under each peak representing the proportion of that fragment size (in bp) in the population. As we know what tract lengths lead to the ON or OFF status of each gene, we can then determine the proportion of the population that is ON or OFF based on this quantification.

Phase variation is the random and reversible switching of gene expression (15). Phase-variable gene expression can occur by several mechanisms, including homologous recombination between allelic variants or variation in the length of simple sequence repeats (SSRs) (15). Phase variation mediated by slipped-strand mispairing of SSRs located within, or associated with, an open reading frame (ORF) commonly leads to the biphasic ON-OFF switching of gene expression (15). This results in the encoded protein being either expressed (ON) or not expressed (OFF) if there was a frameshift mutation, and premature transcriptional termination is introduced (15). The length of SSR tracts has been shown to correlate with rates of phase variation (16–18), with longer tracts exhibiting higher rates of phase variation. The ability to produce multiple phenotypic variants within a bacterial population promotes strain adaptability and survival and allows bacteria to evade host immune responses (15). Lipooligosaccharide (LOS) is a major NTHi virulence factor, and LOS presence has been shown to contribute to survival *in vivo* (19, 20). Many NTHi LOS biosynthetic genes contain SSR tracts and are phase-variably expressed (21, 22). Phase-variable LOS biosynthetic genes include *lic1A*, encoding a phosphorylcholine transferase (23), *lic2A*, encoding a galactosyltransferase (24), *lic3A* and *lic3B*, encoding related sialyltransferases (20, 25), *lex2A*, encoding a glucosyltransferase (26), *lgtC*, encoding a galactosyltransferase (27), and *oafA*, encoding an *O*-acetyltransferase (28) (a summary of NTHi LOS is presented in Fig. 1A). Therefore, ON/OFF switching of the expression of these glycosyltransferases will result in different LOS structures within an NTHi population. We have previously demonstrated that selection for particular LOS biosynthetic genes (*oafA* OFF) occurs with transition from colonizing the human nasopharynx to invading the middle ear cavity during the course of otitis media (19).

Based on previous findings and the importance of LOS in NTHi pathobiology, we hypothesized that the expression of individual LOS biosynthetic gene loci is present or absent, or a particular expression status is selected for (phase-varied ON) or against (phase-varied OFF), during invasive NTHi infection. We used two extensive, unique collections of NTHi taken in South East Queensland, Australia, one containing invasive NTHi isolates collected over 20 years (29) and a second containing nasal swabs from healthy children over the first 2 years of life, the ORChID collection (30). By comparing isolates from the invasive collection to those in the carriage collection, we were able to investigate if differences in LOS structure occurred during invasive disease compared to its structure during carriage. We demonstrate that the expression status of particular LOS biosynthetic genes (*lic2A* and *oafA*) appears to be selected for in invasive NTHi isolates more so than in NTHi carriage isolates.

## RESULTS

By using our fluorescent PCR approach coupled to fragment length analysis, we have been able to determine the ON/OFF expression status of each of seven phase-variable LOS biosynthetic genes (*lic1A*, *lic2A*, *lic3A*, *lic3B*, *lex2A*, *lgtC*, and *oafA*) (Fig. 1) in 70 invasive NTHi isolates collected in South East Queensland, Australia (29). Where PCR products could not be produced for individual genes despite multiple attempts, we analyzed the genome sequences present for invasive isolates (BioProject accession number PRJEB18702) to confirm that these genes were in fact absent from those particular isolates (data not shown). In previous studies of this type, it has also been demonstrated that not all strains contain all seven LOS biosynthetic loci (19). By comparing the ON/OFF expression status of these genes in invasive isolates to that of NTHi carriage isolates from the same region (30), we were able to determine if particular genes are selected for during NTHi invasive infections. Our results show that five of these genes, *lic1A*, *lic3A*, *lic3B*, *lex2A*, and *lgtC*, demonstrated no statistically significant difference for either an ON or an OFF expression state in invasive isolates and did not show a significant difference from the ON/OFF status of carriage isolates. All data from fragment length analysis are presented in Data Set S1 in the supplemental material.

**FIG 2:**
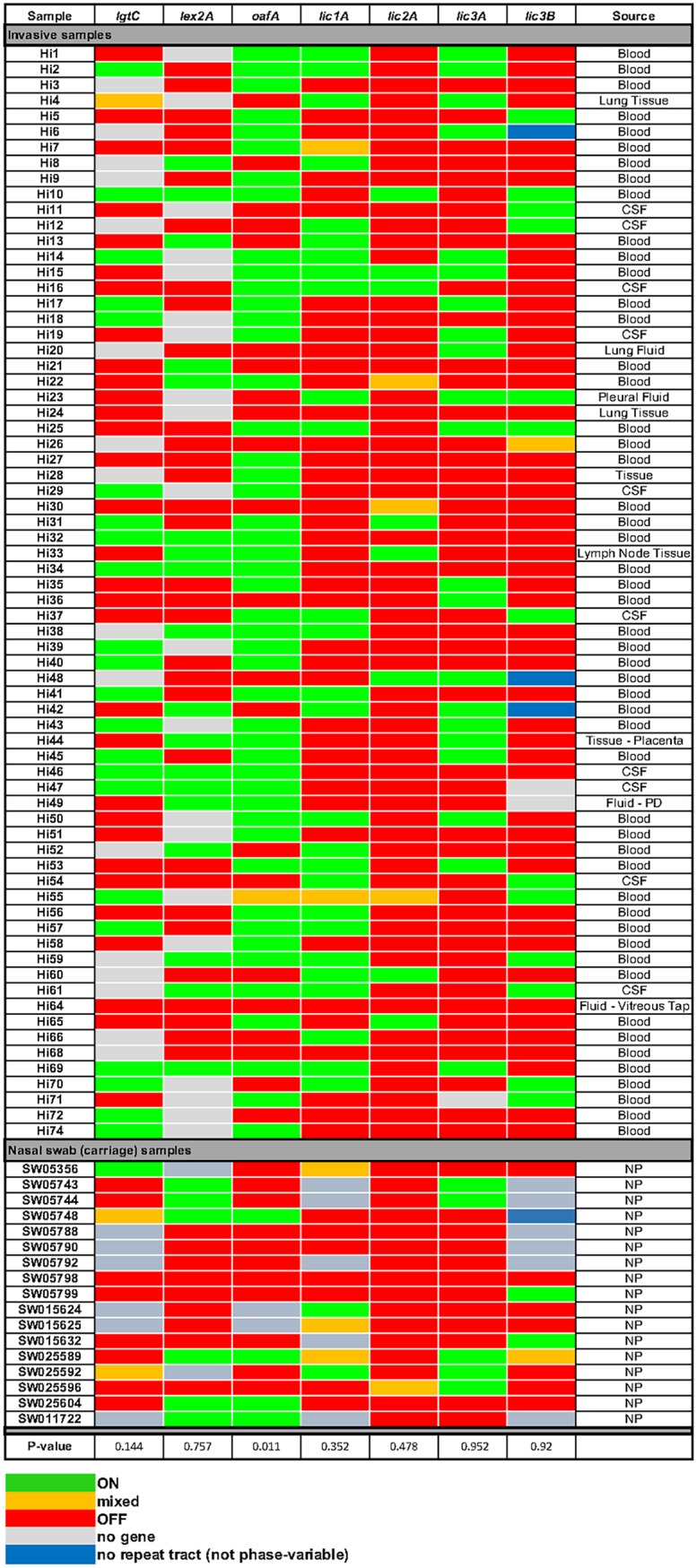
Heat map showing the expression status of each of the seven phase-variable LOS biosynthetic loci assessed in this study. Seventy invasive NTHi isolates (29) and seventeen NTHi carriage isolates (30) were assessed for ON/OFF status using multiplexed fluorescent PCR. Fragment lengths were quantified using an ABI GeneScan system and quantified using PeakScanner software. ON/OFF status was calculated as described previously (19). Green, >70% ON; red, >70% OFF; orange, mixed ON/OFF; blue, no repeat tract; gray, no gene (no product from multiple PCR attempts). All percent ON and OFF values for each collection can be found in Data Set S2. CSF, cerebrospinal fluid; PD, peritoneal dialysis; NP, nasopharynx.

In 59/70 invasive isolates, the *lic2A* gene was OFF, but it is also OFF in the majority of carriage isolates (16/17; no significant difference using a two-tailed Mann-Whitney U test) (Fig. 2). Lic2A is a galactosyltransferase and, in tandem with LgtC, is responsible for the addition of a digalactoside Galα(1-4)βGal moiety (24, 27) onto the LOS. Lic2A activity is responsible for the addition of the first galactose onto a glucose, providing a substrate for LgtC to add the second galactose (Fig. 1A).

We demonstrate that the gene encoding an *O*-acetyltransferase, *oafA*, is generally OFF in carriage isolates but is ON in the majority of invasive NTHi isolates. The *oafA* gene is ON in 47/70 invasive NTHi isolates (67%) but ON in only 4/17 carriage isolates (23%; *P* value of 0.011 using a two-tailed Mann-Whitney U test) (Fig. 2). OafA adds an *O*-acetyl group to the heptose antigen of the inner core of the LOS (Fig. 1A), and it has previously been reported that this O-acetylation, i.e., *oafA* ON, is required for resistance to complement-mediated killing by the host immune system (28). The *oafA* gene is also uniformly present in invasive isolates but is absent from 2/17 carriage isolates. The uniform presence of *oafA* in invasive isolates indicates that all NTHi isolates that are invasive have the potential to switch *oafA* ON.

## DISCUSSION

Our investigation of a large collection of invasive NTHi isolates has allowed us to determine if particular LOS biosynthetic genes are present and have altered expression in sterile niches in the human host. While five out of seven of these biosynthetic genes (*lic1A*, *lic3A*, *lic3B*, *lex2A*, and *lgtC*) show no significant correlation with an ON or OFF expression state during invasive infection, we demonstrate that *lic2A* remains OFF in invasive isolates and *oafA* ON is statistically overrepresented in invasive isolates compared to the level in carriage isolates.

Our observation that *lic2A* is OFF in most invasive isolates is intriguing, as this finding appears contradictory to earlier results. Expression of *lic2A* was previously demonstrated to confer resistance to human serum (31), and modification of the NTHi LOS inner core with a galactose by Lic2A has been shown to shield the cells from *in vitro* neutrophil-mediated killing assays when *lic1A* is phase-varied OFF, with this modification being associated with invasive NTHi isolates (32). However, our findings demonstrate that *lic2A* is OFF in the majority (59/70) of invasive NTHi isolates. Further work is required to identify what factors initially cause Lic2A expression for resistance to serum (*licA2* ON) but then either appear to select against its expression (*licA2* OFF) or do not require its further expression during invasive disease.

We previously demonstrated that *oafA* OFF is selected for during otitis media (19), whereas this work demonstrates *oafA* ON occurs during invasive disease. Previous work with *oafA* expression in NTHi has demonstrated that O-acetylation of the LOS by OafA is required for resistance to complement-mediated killing by human serum (28). The differences in selection for *oafA* expression between two host niches (OFF in the middle ear/ON for invasion and serum resistance) demonstrate the rapid adaptability afforded by phase-variable genes: transition to occupying the middle ear appears to favor *oafA* OFF (19), whereas *oafA* ON occurs during invasive disease and is required for resistance to serum. Interestingly, loss of the related *O*-acetyltransferase OafA in the human enteric pathogen Salmonella enterica serovar Typhimurium, which acetylates the O-antigen of lipopolysaccharide (33), leads to modulation of the immune response and may aid immune evasion (34). Therefore, it appears that acetylation of outer surface oligosaccharides is a common evolutionary mechanism of bacterial pathogens to avoid the immune response and perhaps leads to increased virulence.

Modification of NTHi LOS with other glycan moieties has been shown to be important during pathogenesis. For example, NTHi strains isolated from blood show a decreased phosphorylcholine (PCho) content on their LOS relative to that of nasopharyngeal strains, which leads to decreased binding of antibodies and C-reactive protein (35), which aids survival in blood. However, this study did not investigate if the decreased PCho content of these invasive isolates was due to phase variation of Lic1A, the glycosyltransferase responsible for this modification (Fig. 1). We did not see any switching of *lic1A* in our survey (Fig. 2), which implies that the decreased PCho content of the LOS of invasive isolates (35) is due to a variety of factors that likely includes, but is not absolutely dependent on, *lic1A* switching OFF. Addition of a ketodeoxyoctanoate (KDO) residue as the terminal sugar of LOS rather than *N*-acetylneuraminic acid (Neu5Ac) (Fig. 1) is present during NTHi biofilm formation *in vivo* (36), meaning this modification may cause chronic infection with NTHi. Previous studies examining the role of LOS phase variation in NTHi pathobiology during infection of human volunteers have investigated the ON/OFF status of LOS biosynthetic genes (19, 37) and have shown selection for particular ON/OFF states: *lex2A* and *lic1A* were shown to switch from OFF to ON during nasopharyngeal colonization (37). This *lic1A* finding corroborates the finding that shows decreased PCho in invasive NTHi isolates relative to that of strains from the nasopharynx (35). Our findings that *oafA* ON is selected for during invasive infection, and that the *lic2A* OFF expression state predominates in both carriage and invasive NTHi strains, add an extra level to the complexity of the factors that result in NTHi transitioning from benign carriage to causing overt disease. While we cannot determine if particular LOS structures resulting from the ON/OFF status of these genes lead to invasion or are actually selected for as NTHi moves to particular host niches, i.e., becomes invasive, our work has determined that particular LOS modifications are more prevalent during invasive NTHi disease.

Expression and/or acquisition of particular factors was hypothesized to lead to the emergence of a particularly virulent clone of the closely related organism H. influenzae biogroup *aegyptius* (38), responsible for the acute and fatal invasive infection Brazilian purpuric fever (BPF) (39). +Biogroup *aegyptius* was previously well characterized as a pathogen causing purulent conjunctivitis, but the changes in the organism that were responsible for transition from causing conjunctivitis to causing severe invasive disease are uncharacterized. Nevertheless, several virulence factors were identified (40), with acquisition of particular outer membrane proteins (41), secretion of extracellular proteins (42), expression of certain adhesins (43), and differences in LOS structure (38) all hypothesized to result in BPF, but none were ever conclusively shown to be absolutely required for virulence (38). Our demonstration that *oafA* ON is statistically associated with invasive isolates of NTHi could serve as an indicator for the invasive potential of NTHi strains, and this is one of the first genes shown to be associated with invasive NTHi disease. However, not all invasive isolates in our collection expressed *oafA*, and it is highly likely that there are other uncharacterized factors associated with invasive NTHi infection.

In summary, our work has demonstrated a link between phase variation of particular LOS biosynthetic genes (*oafA* ON and *lic2A* OFF) and invasive disease caused by NTHi. Understanding the expression of these proteins and the structure of LOS during NTHi infection is particularly important, as knowledge of the factors involved in invasive NTHi disease will allow the design of better treatments, allow more accurate diagnosis of infection, and aid in the design of an efficacious and broadly effective vaccine.

## MATERIALS AND METHODS

### Bacterial strains and growth conditions.

Invasive NTHi strains used for this study were isolated from sterile sites in patients suffering H. influenzae infections in South East Queensland over a 15-year period (29). Information on age, site of isolation, and geographical location were all collected, but information on any comorbidity was not (29). The seventy isolates used in this study were selected to represent a broad random sample of the strains present in this collection. NTHi isolates were grown on brain heart infusion (BHI; Oxoid) supplemented (sBHI) with hemin (1%) and NAD (2 μg/ml) at 37°C in an atmosphere containing 5% (vol/vol) CO_2_. Isolates were previously confirmed as NTHi using commercially available sera (Phadebact Haemophilus test; MKL Diagnostics AB, Sollentuna, Sweden, and Denka Seiken, Tokyo, Japan) (29). Whole-genome sequences of each of the seventy isolates were used to perform a BLAST search with NTHi OMP P2 and P6 gene sequences in order to provide additional confirmation (data not shown). Nasal (carriage) control samples were taken from the ORChID collection, a prospective birth cohort study of infants in South East Queensland where daily symptoms were recorded and weekly nasal swabs were collected from 158 infants during their first 2 years of life (30). All samples used as carriage controls are from infants demonstrating no overt symptoms of respiratory illnesses either 2 weeks before or 2 weeks after sampling (44).

### DNA preparation, manipulation, and analysis.

Bacterial genomic DNA from invasive isolates was prepared by boiling a 1-μl loop of each NTHi isolate in 200 μl Tris-EDTA buffer for 20 min, removing the debris by centrifugation (14,000 × *g* for 5 min), and collecting the supernatant, which contained genomic DNA. DNA from the ORChID carriage control samples was isolated as described previously (45). One μl of each DNA preparation was used in each PCR. PCR primers were purchased from Integrated DNA Technologies (IDT; Singapore). Primers are described in Table 1. Multiplex PCR was carried out in 25-μl reaction mixtures using GoTaq DNA polymerase (Promega) according to the manufacturer's instructions. Cycle conditions were the following: initial denaturation at 95°C for 2 min, followed by 30 cycles of denaturation at 95°C for 30 s, annealing at 52°C for 30 s, and extension at 72°C for 30 s, with a final extension at 72°C for 5 min. Samples were checked for multiplex products on 2% (wt/vol) agarose gels buffered with 1× Tris-borate-EDTA. DNA fragments were sized using the GeneScan system (Applied Biosystems International) at the Australian Genome Research Facility (AGRF; Brisbane, Australia), and traces were analyzed using PeakScanner software (Applied Biosystems International). Where a PCR product could not be produced for a particular gene in an isolate, we analyzed the genome sequence available for the invasive collection (PRJEB18702). An illustration of the fragment analysis PCR methodology and an example of a GeneScan trace and PeakScanner quantification are shown in Fig. 1B and C, respectively. The results shown in Fig. 2 indicate whether the genes investigated were ON (>70% ON; green), OFF (>70% OFF; red), or mixed ON and OFF (orange). This was determined from the number of nucleotide repeats in the SSR present in each gene (based on amplicon peak size) and calibrated using previous studies that have demonstrated the relationship between SSR length present in these seven LOS biosynthetic genes and gene expression status (19).

**TABLE 1:** Primers used in this study

Gene	Repeat unit	No. of repeats indicating ON or OFF	Primer sequence^*a*^	Reference
*lgtC*	GACA	10 = ON; 11 and 12 = OFF	For: 5′-VIC-TCATCGAGCAAAGGCATTG-3′	19
			Rev: 5′-CTTACAGCTAAATAAGGTGC-3′	
*lex2A*	GCAA	10 and 11 = OFF; 12 = ON	For: 5′-NED-CGGAATTATGTTAATCAC-3′	19
			Rev: 5′-GTTTGCTTTGTGATGTAC-3′	
*lic2A*	CAAT	10 = ON; 11 and 12 = OFF	For: 5′-FAM-ACTGAACGTCGCAAA-3′	24
			Rev: 5′-GCTAATTAAACAGCCT-3′	
*lic1A*	CAAT	10 and 11 = OFF; 12 = ON	For: 5′-VIC-CAAAAATAACTTTAACGTG-3′	19
			Rev: 5′-AATGCTGATGAAGAAAATG-3′	
*lic3A*	CAAT	10 and 11 = OFF; 12 = ON	For: 5′-NED-ATTACCTGCAATAATGACAG-3′	21
			Rev: 5′-TATTCAATGAACGGTAGAAT-3′	
			Lic3A specific: 5′-GCCAGTAGTCGCAAAAGTGTC-3′	
*lic3B*	CAAT	11 = ON; 12 and 13 = OFF	For: 5′-NED-ATTACCTGCAATAATGACAG-3′	21
			Rev: 5′-TATTCAATGAACGGTAGAAT-3′	
			Lic3B specific: 5′-TCAAACATCTTGCCGTCTTC-3′	
*oafA*	GCAA	9 and 10 = OFF; 11 = ON	For: 5′-FAM-GCCTAATATTTATTATCTCTC-3′	28
			Rev: 5′-GTATGAATAATTAATGCTG-3′	
*modA*	AGCC or AGTC	10 = ON; 11 and 12 = OFF	For: 5′-FAM-ATGGCGGGCAAAGCACCGAAGA-3′	46
			Rev: 5′-CAAAAAGCCGGTCAATTTCATCAAA-3′	

a For, forward; Rev, reverse.

## Supplementary Material

Supplemental file 1

Supplemental file 2
